# Evaluation of genes involved in oxidative phosphorylation in yeast by developing a simple and rapid method to measure mitochondrial ATP synthetic activity

**DOI:** 10.1186/s12934-015-0239-z

**Published:** 2015-04-16

**Authors:** Xiaoting Ye, Kana Morikawa, Shih-Hsin Ho, Michihiro Araki, Keiji Nishida, Tomohisa Hasunuma, Kiyotaka Y Hara, Akihiko Kondo

**Affiliations:** Organization of Advanced Science and Technology, Kobe University, Nada, Kobe 657-8501 Japan; Department of Chemical Science and Engineering, Graduate School of Engineering, Kobe University, 1-1 Rokkodaicho, Nada, Kobe 657-8501 Japan

**Keywords:** Mitochondria purification, ATP synthesis, High-throughput analysis

## Abstract

**Background:**

Measurement of mitochondrial ATP synthesis is a critical way to compare cellular energetic performance. However, fractionation of mitochondria requires large amounts of cells, lengthy purification procedures, and an extreme caution to avoid damaging intact mitochondria, making it the highest barrier to high-throughput studies of mitochondrial function. To evaluate 45 genes involved in oxidative phosphorylation in *Saccharomyces cerevisiae*, we aimed to develop a simple and rapid method to measure mitochondrial ATP synthesis.

**Results:**

To obtain functional mitochondria, *S. cerevisiae* cells were lysed with zymolyase followed by two-step, low- then high-speed centrifugation. Using a firefly luciferin-luciferase assay, the ATP synthetic activity of the mitochondria was determined. Decreasing the ATP synthesis in the presence of mitochondrial inhibitors confirmed functionality of the isolated crude mitochondria. Deletion of genes encoding mitochondrial ATP synthesis-related protein showed their dependency on the oxidative phosphorylation in *S. cerevisiae*.

**Conclusions:**

Compared with conventional procedures, this measurement method for *S. cerevisiae* Mitochondrial ATP Synthetic activity in High-throughput (MASH method) is simple and requires a small amount of cells, making it suitable for high-throughput analyses. To our knowledge, this is the first report on a rapid purification process for yeast mitochondria suitable for high-throughput screening.

## Background

Mitochondria are central organelles controlling the life and death of the cell. They participate in key metabolic reactions, synthesize the majority of ATP in a cell, and regulate a number of signaling cascades, including apoptosis [1]. ATP synthesis is vital for various biological reactions. Many studies have measured ATP content or qualitative changes in total cellular ATP production, but few have quantified ATP production from oxidative phosphorylation of isolated mitochondria [2].

**Figure 1:**
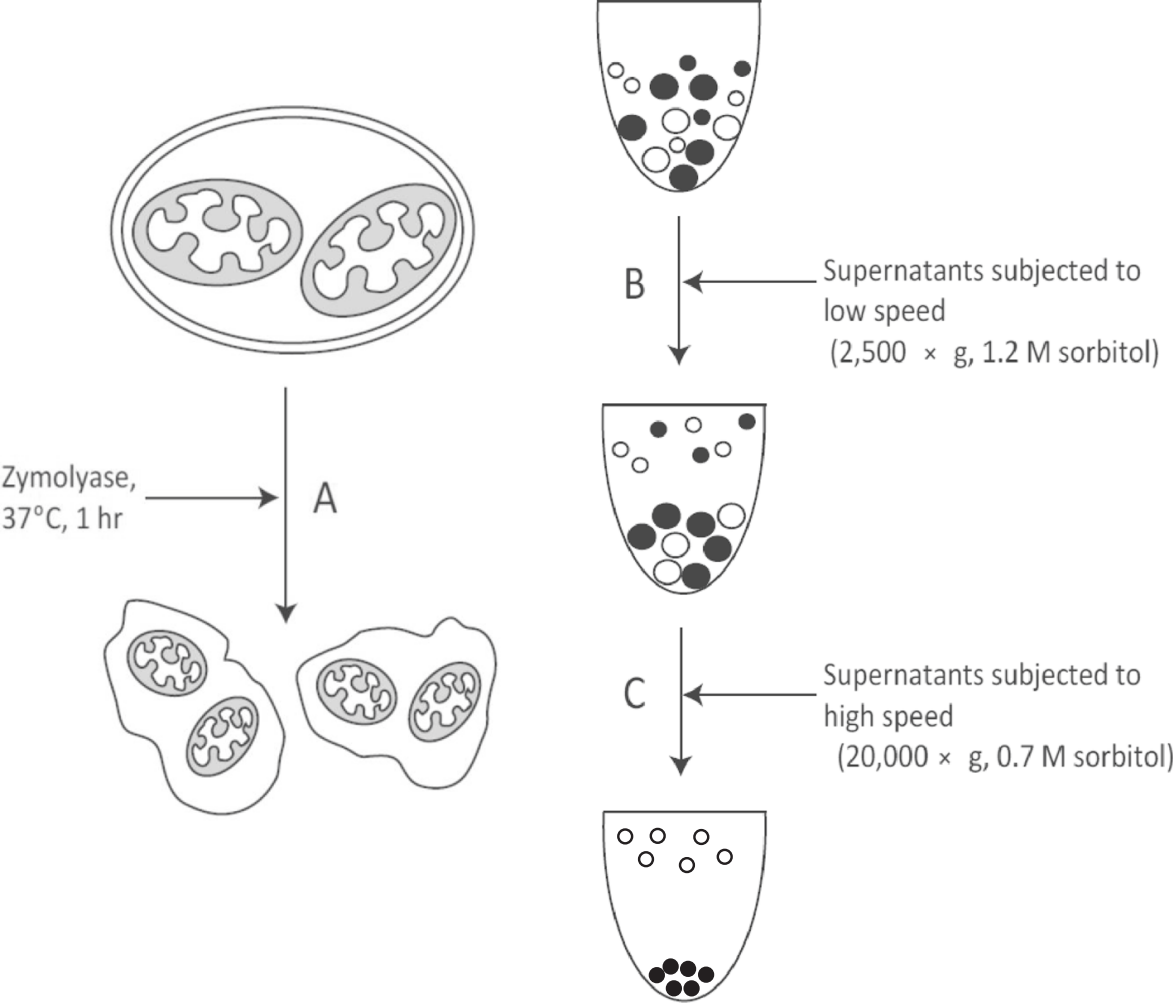
Schematic illustration of the MASH method. **(A)** *S. cerevisiae* cells were lysed with zymolyase at 37°C for 1 h to obtain protoplasts. **(B)** The protoplasts were subjected to low-speed centrifugation (2,500 × *g*) with 1.2 M sorbitol. **(C)** Crude mitochondria were obtained by high-speed centrifugation (20,000 × *g*) with 0.7 M sorbitol. Large circles stand for nuclei, cell debris, and unbroken cells. The small closed-circles stand for the crude mitochondria, the large open-circles stand for microsomes.

Owing to the ease of genetic manipulation and its importance for bio-industry, the budding yeast *Saccharomyces cerevisiae* is an ideal organism for the study of many basic cellular mechanisms in eukaryotic cells. Their organelles can be rapidly enriched in sufficient quantities for the analysis of specific functions such as metabolite or protein transport. Therefore, *S. cerevisiase* is a valuable model cell for studying the molecular and cellular mechanisms underlying the essential biological functions of mitochondria. However, mitochondrial proteins have many subunits, the functions of which are still largely unknown because a method for easy mutational analysis and sensitive assay development is still lacking [3]. One of the biggest problems is that the fractionation of mitochondria requires large amounts of cells, long procedures, and an extreme caution to avoid damaging intact mitochondria [4,5]. In general, to obtain intact mitochondria, the contents of yeast cells are made accessible by a combination of enzymatic digestion of the cell wall and physical disruption of the resulting spheroplasts [6]. To separate the cellular contents by their variable densities, differential centrifugation, which allows for the separation of the constituents of cells based on their different sedimentation properties, is employed to isolate an enriched mitochondrial fraction and is the most common strategy used to obtain crude mitochondria [7]. Crude preparations of mitochondria are contaminated by other organelles such as lysosomes, peroxisomes, tubular Golgi membranes, and, to some extent, small amounts of endoplasmic reticulum. To achieve mitochondria with higher purity, additional time- and labor- consuming purification steps using sucrose density centrifugation are needed. Recently, Frezza *et al.* described a step-by-step method to isolate mitochondria from mouse liver, muscle, and cultured filroblasts using modified differential centrifugation steps and a modified sugar concentration for the osmolyte in the isolation buffer [4]. Based on this technique and protocol for purification of mitochondria from yeast cells [5], we modified this general mitochondria extraction method to quickly obtain crude, but functional mitochondria from yeast cells. The method we developed includes only several steps of differential centrifugation and no sucrose density gradient is needed, which is more suitable for high-throughput screening than the conventional method (Figure 1). By using this method, we evaluated 45 genes involved in oxidative phosphorylation for mitochondrial ATP synthesis in *S. cerevisiae*.

## Results and discussion

### Preparation of crude mitochondria by the MASH method

In the conventional method of mitochondrial purification, yeast cells are subjected to mechanical homogenization or detergent treatment followed by differential centrifugation because the variable density of the organelles will allow separation of the mitochondria from the remaining cellular structures. In the “measurement method for Mitochondrial ATP Synthetic activity in High-throughput” (MASH method) used in the present study, yeast cell walls were lysed with zymolyase (1.2 mg g^−1^ wet cells) at 37°C for 1 h. Zymolyase is an enzyme prepared from *Arthrobacter luteus* that effectively lyses yeast cell wall. The lysis of the cell wall and the formation of the protoplast were verified under a microscope. The crude mitochondria solution was obtained by a two-step, low- (2,500 × *g*) then high- (20,000 × *g*) speed centrifugation with 1.2 M and 0.7 M sorbitol, respectively (Figure 1). Although this suspension is enriched in mitochondria, it may also contain other organelles such as the endoplasmic reticulum, Golgi, and vacuoles. To get more pure mitochondria, this crude mitochondrial fraction can be subjected to further fractionation. However, the crude mitochondria solution obtained using the MASH method is sufficient for the analysis of mitochondrial ATP synthetic activity and therefore was used directly in the ATP assay.

### ATP synthesis with inhibitors of mitochondrial respiratory chain

To confirm the functionality of the mitochondria, several inhibitors of the respiratory chain were used.

**Figure 2:**
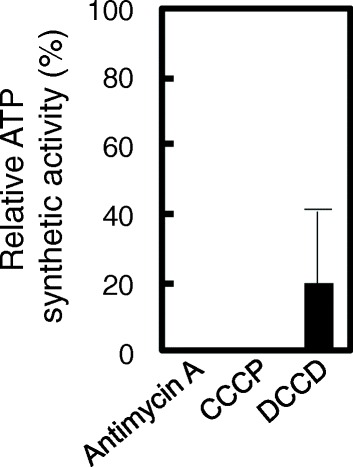
The effect of inhibitors on mitochondrial ATP synthesis in *S. cerevisiae*. Inhibitors of the mitochondrial respiratory chain: antimycin A, CCCP, and DCCD. To confirm the functionality of mitochondria, 0.1 mM antimycin A, 0.5 mM CCCP, and 0.1 mM DCCD were incubated with the crude mitochondria for 5 min before being subjected to ATP assay. The activities were shown in the relative values (%) compared with the value without inhibitor of respiratory chain as the control.

ATP synthesis is inhibited by antimycin A, CCCP, and DCCD [8-11]. Antimycin A, a strong inhibitor of the electron transfer of complex III, functions by binding to the quinone reduction site of the cytochrome *bc*_1_ complex [9]. CCCP is an inhibitor of the proton motive force [10]. DCCD is a specific inhibitor of subunit *c* of complex V (mitochondria F_o_F_1_-ATP synthase) [11]. To test the effect of inhibitors on the mitochondria, antimycin A, CCCP, and DCCD were individually added to the reaction mixture. The addition of either antimycin A or CCCP completely abolished ATP production (Figure 2). The addition of DCCD inhibited ATP production by 80%. These results confirmed that this assay could detect ATP synthetic activity of the crude mitochondria solution using the MASH method and be further applied to high-throughput measurement.

### High-throughput measurement of mitochondrial ATP synthesis

Mitochondrial inhibitors confirmed the functionality of the crude mitochondria isolated using the MASH method. By using this method, the ATP synthetic activities of mutants from the single-gene deletion library of *S. cerevisiae*, including 45 ATP synthesis-related mutants were measured (Table 1). The Saccharomyces Genome Deletion Project created a set of isogenic mutant strains with each individual nonessential gene deleted [12]. This mutant collection has facilitated genome-wide studies to identify genes required for resistance to various cellular insults [13,14]. The set of 45 ATP synthesis-related mutants, which are divided into six genes categories including NADH dehydrogenase (Nde1/Nde2; Complex I), Succinate dehydrogenase (Sdh1b, etc.)/Fumarate reductase (Frd1/Osm1; Complex II), Cytochrome c reductase (Cor1, etc.)/Cytochrome bc_1_ complex (Cbp4, etc.; Complex III), Cytochrome c oxidase (Cbp4, etc.; Complex IV), F_o_F_1_-ATP synthase (Atp1, etc.; Complex V), and others including electron transferring-flavoprotein dehydrogenase (Cir2) and ADP/ATP translocator (Aac1/Aac3). The selected mutant strains and the parental strain were inoculated into 5 ml of YPD medium, grown overnight, and examined for their ability to synthesize ATP using MASH method. The results were shown as the relative value (%) of ATP synthetic activity per mg protein, and the mutant strain values were compared with that of the parental strain. We observed that most of gene deletions in this set resulted in partial loss of the ATP synthetic activity (Table 1).

**Table 1:** **Relative ATP specific activity of gene deletion mutants related to either mitochondrial ATP synthesis or mitochondrial fatty acid synthesis**

**Enzyme**	**Deleted gene**	**Relative ATP specific activity (%)** ^**a**^	**Total protein concentration (mg ml** ^**−1**^ **)** ^**b**^
Parental strain (BY4741)	-	100	0.416
NADH dehydrogenase (Complex I)	*NDE2*	36 ± 4	0.189
	*NDE1*	31 ± 20	0.187
Succinate dehydrogenase/Fumarate reductase (Complex II)	*SDH1b*	44 ± 18	0.189
	*EMI5*	53 ± 41	0.187
	*SDH4*	67 ± 33	0.167
	*SDH1*	33 ± 11	0.181
	*SDH2*	104 ± 39	0.285
	*FRD1*	56 ± 31	0.185
	*OSM1*	61 ± 16	0.207
Electron transferring-flavoprotein dehydrogenase	*CIR2*	56 ± 9	0.195
Cytochrome *c* reductase/Cytochrome *bc*_1_ complex (Complex III)	*COR1*	38 ± 10	0.179
	*RIP1*	37 ± 7	0.179
	*QCR6*	34 ± 21	0.201
	*QCR9*	22 ± 26	0.191
	*QCR10*	72 ± 20	0.187
	*QCR8*	40 ± 9	0.180
	*QCR2*	n.d.	0.177
	*QCR7*	n.d.	0.191
	*CYT1*	n.d.	0.201
	*CBP4*	15 ± 10	0.173
	*FMP25*	29 ± 11	0.203
	*CBP3*	65 ± 25	0.197
	*RCF2*	35 ± 13	0.200
	*CBP6*	27 ± 14	0.190
Cytochrome *c* oxidase (Complex IV)	*COX9*	44 ± 16	0.187
	*COX6*	25 ± 6	0.167
	*COX5B*	37 ± 11	0.191
	*COX12*	20 ± 9	0.193
	*COX8*	62 ± 41	0.175
	*COX7*	n.d.	0.176
	*COX5A*	30 ± 10	0.182
ATP synthase (Complex V)	*ATP1*	32 ± 12	0.177
	*ATP2*	18 ± 5	0.234
	*ATP7*	52 ± 28	0.179
	*ATP14*	52 ± 29	0.187
	*ATP20*	17 ± 8	0.189
	*ATP18*	40 ± 16	0.168
	*ATP4*	98 ± 73	0.206
	*ATP15*	24 ± 16	0.197
	*ATP5*	38 ± 4	0.187
	*INH1*	43 ± 14	0.175
	*STF1*	43 ± 17	0.171
	*STF2*	53 ± 10	0.190
ADP/ATP translocator	*AAC1*	59 ± 7	0.178
	*AAC3*	53 ± 24	0.208

^a^Relative ATP specific activity of the mutant strains was expressed as a percentage of the specific activity of the parental strain.

^b^The protein concentration of the crude mitochondria solution was determined using the Bradford method [25].

All experiments were conducted at least in triplicate. Values are means ± SD.

Among the mutant strains examined, deletion of genes directly related to the mitochondria respiratory chain (NADH dehydrogenase, succinate dehydrogenase, electron transferring-flavoprotein dehydrogenase, cytochrome *c* reductase, cytochrome *bc*_1_ complex, cytochrome *c* oxidase, and F_o_F_1_-ATP synthase) showed lower ATP synthetic activity compared with that of the parental strain, indicating that these components of the mitochondria respiratory chain were indispensable for ATP synthetic activity.

In the case of F_o_F_1_-ATP synthase (complex V), 12 single gene deletion strains were used to measure their ATP synthetic activity by the MASH method. Deletion of *ATP4* had almost no effect on the ATP synthetic activity because *ATP4* encodes b subunit which effects on the stability of oligomeric F_o_F_1_-ATP synthases, not ATP synthetic activity [15]. As the result, especially in both the Δ*ATP2* strain and Δ*ATP20* strain, the ATP synthetic activities were drastically decreased compared to the other mutant strains lacking gene encoding a subunit of the F_o_F_1_-ATP synthase. Their ATP synthetic activities were 20% of that of the parental strain. *ATP2* and *ATP20* encode β subunit and γ subunit of the F_o_F_1_-ATP synthase, respectively. The β subunit is the catalytic subunit of F_o_F_1_-ATP synthase and the γ subunit constructs a stalk structure connecting the proton-motive force generated in F_o_-part and ATP synthesis in F_1_-part of F_o_F_1_-ATP synthase [16]. Thus, the deletions of these functionally important genes, *ATP2* and *ATP20*, indicate completely loss of ATP synthesic ability of F_o_F_1_-ATP synthase. These reasonable results guarantee the validity of this assay. The remaining 20% activities of ATP synthesis compared to the parental strain in the Δ*ATP2* strain and Δ*ATP4* strain indicate the ATP synthesis by mitochondrial adenylate kinase (2ADP → AMP + ATP) encoded by *ADK2* [17]. Some deletion mutants (*QCR2*, *QCR7*, *CYT1*, *CBP4* and *COX7*) showed lower ATP synthetic activities rather than 20% of the parental strain. This result indicates that the deletion of these genes enhance the hydrolysis of ATP resulted from adenylate kinase. The ATP hydrolysis would be catalyzed through reversible reaction of ATP synthesis by F_o_F_1_-ATP synthase because of the lower H^+^-gradient formed between inside and outside of mitochondrial inner membrane.

Aside from the respiratory chain mutants, the ADP/ATP translocator, which is mainly responsible for transferring ADP/ATP in or out of the mitochondria [18], was also tested. Notably, deletion of genes *AAC1* and *AAC3* encoding the ADP/ATP translocator, resulted in a 50% - 60% loss of ATP synthetic activity compared with that of the parental strain. Previous studies demonstrated that disruption of *AAC1* or *AAC3* did not influence the content of the ADP/ATP translocator, and both *AAC1* and *AAC3* genes did not substantially participate in mitochondrial ADP/ATP transport under normal growth conditions [19]. On the contrary, our result demonstrated that *AAC1* and *AAC3* caused some loss of ATP synthetic activity, indicating that although these genes may not be directly involved in mitochondrial ADP/ATP transport, they are potentially responsible for mitochondrial ATP synthesis. Further elucidation of the underlying mechanism is needed.

When deleted, three genes (*QCR2*, *QCR7*, and *CYT1*) encoding subunits of cytochrome *c* reductase (complex III) have been shown to have undetectable ATP synthetic activity (Table 1). Cytochrome *c* reductase is essential to the energy-generating process of oxidative phosphorylation [20]. Qcr2 is one of the core subunits of complex III, and its mutation has been demonstrated to cause either a severe decrease or a total block in complex III activity and respiratory growth [21]. Cyt1 (cytochrome *c*_1_) is one of the catalytic subunits of the cytochrome *bc*_1_ complex and is essential for electron transfer and for the respiratory growth [22]. Therefore, the deletion of Qcr2 and Cyt1 led to dramatically decreased ATP synthetic activity. Together with the result that the addition of antimycin A completely suppressed ATP synthetic activity (Figure 1), this result further indicated that cytochrome *c* reductase played an important role in mitochondrial ATP synthesis.

**Figure 3:**
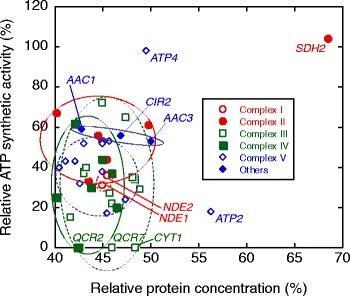
Distribution of relative protein concentration and relative ATP synthetic activity of crude mitochondria solutions from gene deletion mutants related to mitochondrial ATP synthesis. The relative protein concentrations of the crude mitochondria solutions were determined using the Bradford method. Relative ATP synthetic activity was normalized by each protein concentration of the mutant strains. These values are expressed as a percentage of its activity of the parental strain. The relative ATP synthetic activities under detectable level were plotted at “zero”. All experiments were conducted at least in triplicate and values are represented as means.

To further test the feasibility of the MASH method for measuring ATP synthetic activity, we compared the distribution of protein concentration and ATP synthetic activity in crude mitochondrial solutions from 45 single-gene deletion strains related to mitochondrial ATP synthesis. The relative protein concentration (%) versus relative ATP synthetic activity (%) of each strain compared to the parental strain was plotted (Figure 3). This distribution map indicated that the relative protein concentration and the ATP synthetic activity were not correlated. All tested strains were distributed in the 40-50% range for relative protein concentration except the *ATP2* and *SDH2* deletion strains. In contrast, these strains were broadly distributed for the relative ATP synthetic activity. Strains lacking *NDE1* or *NDE2* involved in Complex I were plotted near to each other. Deletion mutants for genes involved in Complex II were distributed in the 30-70% range except the *SDH2* deletion strain. Strains lacking genes involved in Complex III and IV were broadly distributed in the 0-72% and 0-62% ranges, respectively. In contrast, deletion mutants for genes involved in Complex V were narrowly distributed in the 18-53% range except the *ATP4* deletion strain, which showed almost the same ATP synthetic activity as the parental strain. Deletion of all three genes involved in other proteins related to ATP synthesis including electron transferring-flavoprotein dehydrogenase (*CIR2*) and two ADP/ATP translocators (*AAC1* and *AAC3*) showed almost 55% of the relative ATP synthetic activity compared to the parental strain.

This result demonstrates the wide applicability of the MASH method. As shown in this study, the MASH method can propose new areas of study to resolve the cellular ATP synthesis mechanism.

### Potential applications of the MASH method

The MASH method is a simple and rapid way to obtain a crude mitochondria solution and determine respiratory ATP synthesis in yeast cells. Mitochondria have been isolated from yeast using the combination of zymolyase and Dounce homogenization for many years at least since 1982 from the Schatz’s laboratory [7]. Thus, in the conventional methods, to prepare intact mitochondria from yeast, cells are collected, then disrupted by mechanical homogenization or detergent treatment. Next, the suspension is separated using differential centrifugation, and the fraction containing mitochondria is subjected to several steps of differential gradient centrifugation, which takes 4–5 hours. In contrast, the MASH method can be finished within two hours and is free of contamination that affects the ATP activity assay, simplifying the purification procedure. Moreover, because the method needs only a small amount of cells and has no requirement for retrieving the band containing mitochondrial fraction from the centrifuged gradient, it is suitable for high-throughput (*e.g.,* 96-well format) analysis of mutants and drugs.

It is worth noting that any components whose defects result in loss of mitochondrial ATP synthesis can be measured by the MASH method. By using a combination of specific mitochondrial inhibitors and single gene deletion mutant strains, the point of defect could be determined. Furthermore, if the original or mutated target genes are added back into the knockout cells by transformation, their function can be investigated in more detail. One application of this method would be a functional test for activities of the respiratory chain complexes I, II, III, IV, V, the ADP/ATP translocator, and other ATP synthesis-related proteins in mitochondria.

## Conclusion

This method is designated as measurement method for *S. cerevisiae* Mitochondrial ATP Synthetic activity in High-throughput (MASH method). To obtain crude, yet functional mitochondria, only three steps are included (Figure 1). ATP production from isolated mitochondria could be determined by a bioluminescence assay. By using this MASH method, systematic analysis of gene deletion mutants related to the mitochondrial ATP synthesis was carried out to identify genes required for ATP synthesis, providing a global view of these genes in maintaining ATP activity. The MASH method described in this study introduces a quick and reproducible methodology for measuring ATP synthetic activity in isolated mitochondria.

## Methods

### Yeast strains and growth conditions

The parental strain *S. cerevisiae* BY4741 (*MAT*α *his3∆1 leu2∆0 met15∆0 ura3∆0*) was cultured in 5 ml of YPD medium containing 10 g L^−1^ yeast extract, 20 g L^−1^ bacto-peptone, and 20 g L^−1^ glucose overnight at 30°C. The collection of yeast knockouts was purchased from Invitrogen. Zymolyase-20T was purchased from Seikagaku Kogyo Co. (Tokyo, Japan). Protease inhibitor cocktail and D-luciferin was purchased from Roche (Basel, Switzerland). Firefly luciferase was obtained from Promega (Madison, WI, USA). Other chemicals were purchased from Nacalai Tesque (Kyoto, Japan) or Wako Chemicals (Osaka, Japan).

### Measurement of ATP synthetic activity

All solutions were stored at 4°C and freshly prepared prior to use. Cultures of *S. cerevisiae* cells were grown aerobically in 5 ml of YPD medium at 30°C for 24 h. The optical density at 600 nm (OD_600_) was measured using a UVmini-1240 spectrophotometer (Shimadzu, Kyoto, Japan). The cells were collected, washed with 0.5 ml of 10 mM EDTA, and centrifuged (400 × *g*, 5 min, 4°C). The supernatant was discarded, and the pellets were resuspended in 50 mM Tris–HCl (pH 7.5), 1.2 M sorbitol, 10 mM EDTA, 0.3% (v/v) 2-mercaptoethanol, and 1.2 mg g^−1^ wet cells of the zymolyase solution (4 mg ml^−1^). After incubation at 37°C for 1 h with rotary agitation, the lysis of the cell wall was verified under a microscope. The supernatant was resuspended in 50 mM Tris–HCl (pH 7.5), 0.7 M sorbitol, 10 mM EDTA, 1 mM PMSF, protease inhibitor cocktail, and 20 mM triethanolamine, and then subjected to the low-speed centrifugation step (2,500 × *g*, 15 min, 4°C). The debris was discarded and the supernatant was then subjected to the high-speed centrifugation step (20,000 × *g*, 15 min, 4°C). The resulting pellets obtained were dissolved in 50 mM Tris–HCl buffer (pH 7.5), and stored at 4°C before use.

The ATP assay was conducted as previously described [23,24]. The reaction buffer containing 50 mM Tris–HCl (pH 7.5), 1.3 μg ml^−1^ luciferase, 0.05 mM D-luciferin, 1 mM DTT, 5 mM MgCl_2_, and 0.1 mM EDTA was added to the crude mitochondria solution. The reaction was initiated by addition of 0.1 mM ADP, and the luminescence was measured using EnVision Multilabel Reader 2104 (PerkinElmer, Waltham, MA, USA). The luminescence of each well was measured at 1 s intervals. The ATP synthetic activities of the crude mitochondria solutions were calculated by taking away the background luciferase activity in the presence of ADP. The relative ATP synthetic activity was normalized each protein concentration determined using the Bradford method [25] of the mutant strains. The values are expressed as a percentage of its activity of the parental strain. To test the functionality of mitochondria, the inhibitors (0.1 mM antimycin A, 0.5 mM CCCP, and 0.1 mM DCCD) were incubated with the crude mitochondria solution for 5 min before being subjected to the ATP assay.

### High-throughput measurement for mitochondrial ATP synthesis

The glycerol stock of yeast gene-deletion mutants was inoculated with a sterilized toothpick to 5 ml of YPD medium. Cells were cultured overnight at 30°C with shaking. The cells were harvested by centrifugation (3,000 × *g*, 10 min, 4°C) and washed twice with distilled water. The purification of mitochondria was conducted as described above. Protein concentration was measured with the Bio-Rad assay system (Bio-Rad, Hercules, CA, USA) using bovine serum albumin as the standard. Relative specific activity (%) was calculated from the ratio of total activity divided by total protein concentration of the mutant strain to that of the parental strain. Z’ of this assay can be calculated as 0.58.

## Abbreviations

ATP: Adenosine 5′-triphosphate
G3P: Glycerol 3-phosphate
CCCP: Carbonylcyanide
DCCD: *N,N′*-dicyclohexylcarbodiimide
pmf: Proton motive force
NADH: Nicotinamide adenine dinucleotide hydrogen
ADP: Adenosine-5′-diphosphate
EDTA: Ethylenediaminetetraacetic acid
Tris: 2-Amino-2-hydroxymethyl-propane-1,3-diol(hydroxymethyl)aminomethane
PMSF: Phenylmethylsulfonyl fluoride
DTT: Dithiothreitol

## References

[1] Stump CS, Short KR, Bigelow ML, Schimke JM, Nair KS (2003). Effect of insulin on human skeletal muscle mitochondrial ATP production, protein synthesis, and mRNA transcripts. Proc Natl Acad Sci U S A.

[2] Drew B, Leeuwenburgh C (2003). Method for measuring ATP production in isolation mitochondria: ATP production in brain and liver mitochondria of Fischer-344 rats with age and caloric restriction. Am J Physiol Regul Integr Comp Physiol.

[3] Fujikawa M, Yoshida M (2010). A sensitive simple assay of mitochondria ATP synthesis of cultured mammalian cells suitable for high-throughput analysis. Biochem Biophys Res Commun.

[4] Frezza C, Cipolat S, Scorrano L (2007). Organelle isolation: functional mitochondria from mouse liver, muscle and cultured filroblasts. Nat Protoc.

[5] Gregg C, Kyryakov P, Titorenko V (2009). Purification of mitochondria from yeast cells. J Vis Exp.

[6] Granham JM. Purification of a crude mitochondrial fraction by density-gradient centrifugation. Curr Protoc Cell Biol. 2001, doi:10.1002/0471143030.cb0304s04. 10.1002/0471143030.cb0304s04 18228356

[7] Daum G, Böhni PC, Schatz G (1982). Import of proteins into mitochondria. Cytochrome b2 and cytochrome c peroxidase are located in the intermembrane space of yeast mitochondria. J Biol Chem.

[8] Wibom R, Lundin A, Hultman E (1990). A sensitive method for measuring ATP-formation in rat muscle mitochondria. Scand J Clin Lab Invest.

[9] Han YH, Kim SH, Kim SZ, Park WH (2008). Antimycin A as a mitochondrial electron transport inhibitor prevents the growth of human lung cancer A549 cells. Oncol Rep.

[10] Makowska A, Zablocki K, Duszynski J (2000). The role of mitochondria in the regulation of calcium influx into Jurkat cells. Eur J Biochem.

[11] Yamamoto T, Terauchi S, Tachikawa A, Yamashita K, Kataoka M, Terada H (2005). Two critical factors affecting the release of mitochondrial cytochrome *c* as revealed by studies using N, N’-dicyclohexylcarbodiimide as an atypical inducer of permeability transition. J Bioenerg Biomembr.

[12] Huang ME, Rio AG, Nicolas A, Kolodner RD (2003). A genomewide screen in *Sacchromyces cerevisiae* for genes that suppress the accumulation of mutants. Proc Natl Acad Sci U S A.

[13] Birrell GW, Brown JA, Wu HI, Giaever G, Chu AM, Davis RW (2001). Transcriptional response of *Saccharomyces cerevisiae* to DNA-damaging agents does not identify the genes that protect against these agents. Proc Natl Acad Sci U S A.

[14] Chan TF, Carvalho J, Riles L, Zheng XF (2000). A chemical genomics approach toward understanding the global functions of the target of rapamycin protein (TOR). Proc Natl Acad Sci U S A.

[15] Weimann T, Vaillier J, Salin B, Velours J (2008). The intermembrane space loop of subunit b (4) is a major determinant of the stability of yeast oligomeric ATP synthases. Biochemistry.

[16] Hara KY, Kato-Yamada Y, Kikuchi Y, Hisabori T, Yoshida M (2001). The role of the betaDELSEED motif of F_1_-ATPase: propagation of the inhibitory effect of the epsilon subunit. J Biol Chem.

[17] Gu Y, Gordon DM, Amutha B, Pain D (2005). A GTP:AMP phosphotransferase, Adk2p, in *Saccharomyces cerevisiae*. Role of the C terminus in protein folding/stabilization, thermal tolerance, and enzymatic activity. J Biol Chem.

[18] Klingenberg M (2008). The ADP and ATP transport in mitochondria and its carrier. Biochim Biophys Acta.

[19] Drgon T, Sabova L, Nelson N, Kolarov J (1991). ADP/ATP translocator is essential only for anaerobic growth of yeast *Saccharomyces cerevisiae*. FEBS.

[20] Dorsman JC, Grivell LA (1990). Expression of the gene encoding subunit II of yeast QH2: cytochrome *c* oxidoreductase is regulated by multiple factors. Curr Genet.

[21] Oudshoorn P, Van Steeg H, Swinkels BW, Schoppink P, Grivell LA (1987). Subunit II of yeast QH2: cytochrome *c* oxidoreductase. Nucleotide sequence of the gene and features of the protein. Eur J Biochem.

[22] Hunte C, Palsdottir H, Trumpower BL (2003). Protonmotive pathways and mechanisms in the cytochrome *bc*1 complex. FEBS Lett.

[23] Hara KY, Mori H (2006). An efficient method for quantitative determination of cellular ATP synthetic activity. J Biomol Screen.

[24] Hara KY (2009). Permeable cell assay: a method for high-throughput measurement of cellular ATP synthetic activity. Methods Mol Biol.

[25] Bradford MM (1976). A rapid and sensitive method for the quantitation of microgram quantities of protein utilizing the principle of protein-dye binding. Anal Biochem.

